# Enhanced expression of TWIK-related arachidonic acid-activated K^+^ channel in the spinal cord of detrusor overactivity rats after partial bladder outlet obstruction

**DOI:** 10.1186/s12894-015-0092-8

**Published:** 2015-10-06

**Authors:** Junlong Zhang, Mingxin Cao, Xilian Wu, Yu Chen, Weijie Liang, Yueyou Liang

**Affiliations:** Department of Urology, The First Affiliated Hospital of Sun Yat-Sen University, NO. 58 Zhongshan Er Road, Guangzhou, 510080 China; Department of Urology, HuiZhou Affiliated Hospital, Sun Yat-sen University (HuiZhou Municipal Central Hospital), Huizhou, Guangdong, China; Department of Urology, The Eastern Hospital of the First Affiliated Hospital, Sun Yat-Sen University, Guangzhou, China

**Keywords:** Detrusor overactivity, Partial bladder outlet obstruction, Rat, Spinal cord, TWIK-related arachidonic acid-activated K^+^ channel

## Abstract

**Background:**

Detrusor overactivity (DO) secondary to partial bladder outlet obstruction (PBOO) is closely associated with alteration of ion channels. The objective of this study is to investigate the expression of the TWIK-related arachidonic acid-activated K^+^ channel (TRAAK) in the L6-S1 spinal cord of DO rats after PBOO.

**Methods:**

Female Sprague–Dawley rats undergoing PBOO surgery were screened for DO by cystometry. Sham-operated rats served as controls. The expression of TRAAK in the L6-S1 spinal cord was detected by real-time polymerase chain reaction, western blotting and immunohistochemistry.

**Results:**

DO was successfully induced after chronic PBOO in rats, with an incidence rate of 62.5 %. Compared with sham-operated rats, the expression of TRAAK in the L6-S1 spinal cord of DO rats was significantly increased at the mRNA (1.886 ± 0.710 versus 0.790 ± 0.679, *P* < 0.05) and protein level (0.510 ± 0.087 versus 0.255 ± 0.107, *P* < 0.05). Immunohistochemical staining showed increased expression of TRAAK in the dorsal horn and ventral horn of the spinal cord.

**Conclusions:**

Upregulation of TRAAK was observed in the spinal cord of DO rats after chronic PBOO, which may exert a protective effect against DO by suppressing the excitability of neurons.

## Background

Detrusor overactivity (DO) is highly correlated with overactive bladder symptoms and commonly occurs in combination with bladder outlet obstruction [1]. It is reported that DO was present in 61 % of patients with lower urinary tract symptoms attributed to benign prostatic obstruction in man [2]. Several types of ion channels, including the T-type calcium channel and calcium-activated K^+^ and Cl^−^ channels, have been showed to be activated or suppressed in the bladder in animal models with DO induced by partial bladder outlet obstruction (PBOO) [3, 4] and play a critical role in generating spontaneous activity in the detrusor muscle through a myocyte mechanism [5–7]. However, most studies on ion channels have only focused on the myocyte mechanism of detrusor muscle activation. Very few studies have investigated the effect of PBOO on the biochemical status of the central nervous system. A recent study found that the expressions of T-type Ca^2+^channels and N-type Ca^2+^ channels were up-regulated in the spinal cord dorsal horn of rats with bladder outlet obstruction induced by partial urethral ligation [8], which suggests that bladder outlet obstruction not only influences the bladder wall, but also the central nervous system, which is distant from the bladder.

K^+^ channels play an important role in cell functions and consist of three classes. The Weak inward rectifying K^+^ channel (TWIK) is one class of K^+^ channels that includes four transmembrance segments and two pore domains. The TWIK-related K^+^ channel (TREK) subfamily, including the TWIK-related arachidonic acid-activated K^+^ (TRAAK), TREK-1 and TREK-2 channels, has been shown to be associated with resting membrane potential and cellular excitability [9]. Previous works by our group showed that the expression of the TRAAK channel was down-regulated in the L6-S1 spinal cord of rats with complete bladder outlet obstruction (CBOO) [10]. This downregulation of the TRAAK channel was thought to enhance the excitability of neurons and increase the sensitivity of the bladder. In PBOO rats, the TREK-1 channel has been found to be down-regulated in detrusor myocytes of the bladder, and this was thought to be associated with bladder overactivtity [11]. However, alteration in TRAAK channel expression in the central nervous system in PBOO rats has never been explored, and it may regulate the excitability of neurons and, subsequently, be associated with DO. Therefore, the present study investigated TRAAK channel in the spinal cord of a PBOO-induced DO rat model.

## Methods

### Animals

The experimental protocol was approved by the animal ethics committee of Sun Yat-Sen University. All experimental procedures were conducted according to the guidelines for animal experiments. Thirty female Sprague–Dawley rats weighting 200–220 g were randomly divided into sham-operated control and PBOO groups. All animals were kept in mesh-bottom cages with a 12 h light/12 h dark cycle, the temperature maintained at 22–24 °C, and free access to food and water.

### Preparation of PBOO models

The PBOO model was established according to the report of Mattiasson A [12]. All animals were anesthetized with urethane (1 g/kg, i.p.). After a low abdominal incision, the proximal urethra was tightly ligated using a 2/0 silk ligature, and a small plastic tube (O.D. 1.0 mm) was placed as a catheter via the urethral orifice. The tube was then removed, and the abdominal incision was closed. Sham-operated rats underwent the same procedure without ligature. After operation, the animals received prophylactic antibiotics treatment of 20,000 units of penicillin for 3 days.

### Cystometry and group classification

Six weeks after PBOO, filling cystometry was performed on all rats. The procedure was performed according to previous reports [10]. After anesthetization, the bladders of the rats were exposed through the incision of the PBOO surgery. The dome of the bladder was punctured with a 22-G angiocatheter, and it was connected to a MR-301 single-path syringe pump (MeiRuiHua Medical technology, Zhuhai, China). To measure the intravesicular pressure, a BL-420E+ Data Acquisition & Analysis system (Chengdu TME Technology, Chengdu, China) was connected to the syringe pump via a 3-way stopcock. While infusing warm saline (37–38 °C) at 0.2 ml/min, intravesicular pressure was monitored. According to the cystometry result, PBOO rats displaying nonvoiding contractions (NVCs) before the onset of micturition were selected as the DO group. PBOO rats exhibiting stable detrusor function before the onset of micturition were excluded from the experiment. Sham-operated rats without NVCs were classified as the control group.

### Tissue preparation

After cystometry, rats were anesthetized with an overdose of urethane (4 g/kg, i.p.). For real-time polymerase chain reaction (RT-PCR), 7 rats in the DO group and 7 rats in the sham-operated group were perfused transcardially with ice-cold heparinized saline for 10 min. Then, the bladder and L6-S1 spinal cord were collected and placed in liquid nitrogen. For immunohistochemical staining, 3 rats in the DO group and 3 rats in the sham-operated group were perfused transcardially with heparinized saline for 10 min, followed by 4 % paraformaldehyde for 20 min. The bladder and L6-S1 spinal cord were embedded in paraffin after being post-fixed for 3 days in 4 % paraformaldehyde. Paraffin-embedded tissues were cut into 5-μm-thick sections and stored at −80 °C. The bladders of the rats were weighed after collection.

### Quantitative RT-PCR

Using Trizol reagent (Life Technologies, Karlsruhe, Germany), the total RNA of the bladder and L6-S1 spinal cords of the rats were extracted from 100 mg of the tissue. The concentration and quality of RNA were determined by ultraviolet spectrophotometry. Total RNA was reverse transcribed into cDNA using a ReverTra Ace® qPCR RT Kit (TOYOBO). RT-PCR was performed using SYBR® Premix Ex TaqTM with a Mastercycler® ep realplex Real Time system. The sequences of special the primer pair used for TRAAK were as follows: AACTCGCGCAGAGATGGGTGG (forward) and AGGGCAGGAGTGGTTGCTCCT (reverse). The cycle conditions included 30 s at 95 °C, followed by 40 cycles of denaturation at 95 °C for 5 s every 1 min and annealing at 60 °C for 30 s. We used β-actin expression as an endogenous control.

### Western blotting

Four rats from each group were randomly selected for western blotting. Tissues were dissected and homogenized in RIPA buffer containing protease inhibitors. After homogenization, the proteins were subjected to 12 % SDS-PAGE. The protein bands were transferred to polyvinylidene fluoride membranes. Then, the membranes were incubated with TRAAK antibody (dilution 1:1000; Alomone Labs, Jerusalem, Israel) for 3 h at room temperature, followed by incubation with horseradish peroxidase-conjugated secondary antibody (dilution 1:5000) for 1 h at room temperature. The blots were visualized using the ECL system (Millipore, Bedford, USA). Equal protein loading was confirmed by measurement of β-actin.

### Immunohistochemistry

For immunohistochemistry, the sections were incubated with 0.3 % H_2_O_2_ to block endogenous peroxidase activity and were incubated with bovine serum albumin for 1 h to block nonspecific protein binding. Then, the sections were incubated overnight at 4 °C with TRAAK polyclonal antibody (dilution 1:100, Alomone Labs) as the primary antibody and then with secondary antibody for 30 min at 37 °C. The immunoreaction products were visualized using 3,3'-diaminobenzidine tetrahydrochloride. The sections were counterstained with hematoxylin, dehydrated in gradient alcohol, and cleared in xylene. For the negative control, 0.01 mol/L PBS was used in place of the primary antibody. The immunohistochemistry results were evaluated by two researchers blinded to the group allocation. The distribution and amount of positive cells were compared between the DO and sham-operated groups.

### Statistical analysis

Data are presented as means ± SD. The Students’ *t*-test was used for comparison between groups. *P* < 0.05 was considered to indicate a statistically significant difference. All statistical analyses were performed using the SPSS version 17.0 Statistic software package (IBM, New York, NY, USA).

## Results

### Mortality rate and cystometry

**Fig. 1:**
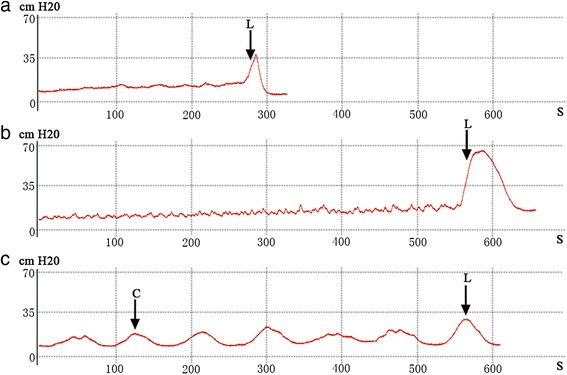
Examples of cystometrograms of the three groups. **a** sham-operated group, **b** non-DO group, **c** DO group. C = NVC; L = leakage of urine from the urethra orifice

After operation, two PBOO rats died due to severe bladder outlet obstruction. One PBOO rat and one sham-operated rat died of infection. Cystometry showed that 10 out of 16 (incidence rate 62.5 %) PBOO rats displayed NVCs before the onset of micturition (Fig. 1). These rats were classified as the DO group. None of the sham-operated rats exhibited NVCs before the onset of micturition, and they were classified as the control group. Compared with the sham-operated rats, the DO rats had significantly greater bladder capacity (12.82 ± 4.47 versus 0.84 ± 0.34 ml, *P* < 0.001) and more post-void residual urine volume (9.78 ± 3.14 versus 0.22 ± 0.17 ml, *P* < 0.001).

### Body and bladder weight

Six weeks after PBOO operation, no significant differences in body mass were observed between the DO rats and sham-operated rats (270 ± 24 versus 270 ± 35 g, *P* > 0.05). However, the bladder wet weights of the DO rats were significantly greater than those of the sham-operated rats (0.760 ± 0.222 versus 0.178 ± 0.087 g, *P* < 0.001).

### TRAAK mRNA expression

**Fig. 2:**
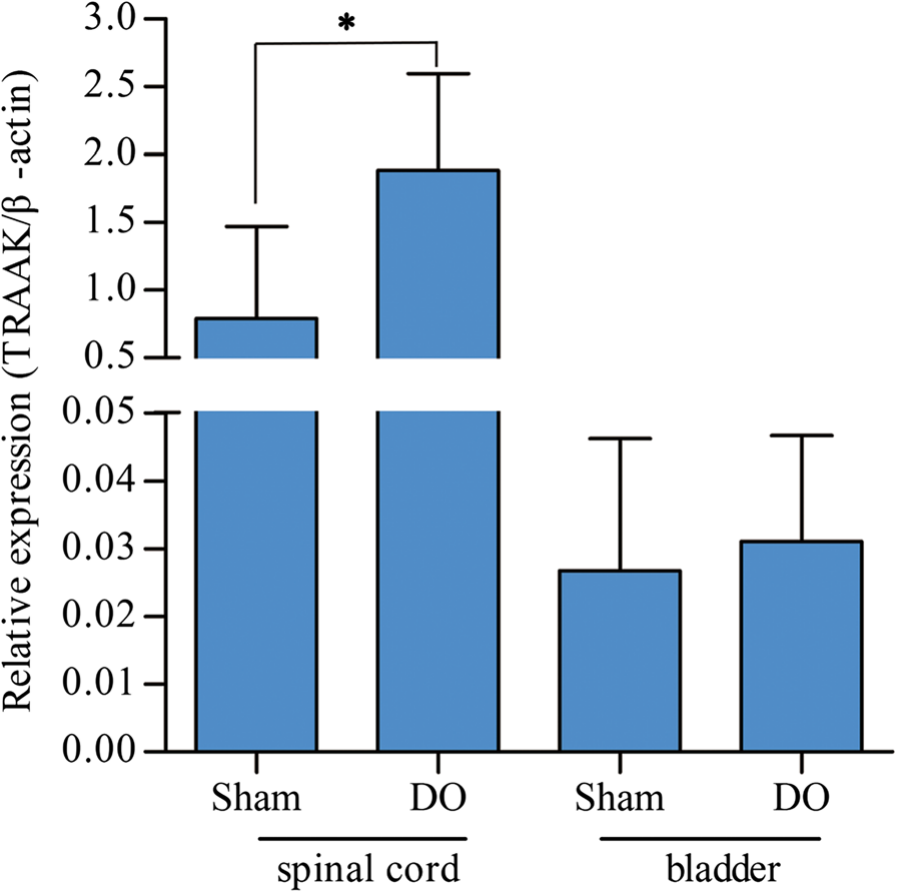
TRAAK mRNA expression. TRAAK mRNA expression in the L6-S1 spinal cords and bladders of DO and sham-operated rats were normalized to β-action. Data are presented as means (SD) of TRAAK/β-action. DO rats *n* = 7; Sham-operated rats *n* = 7. *P < 0.05

As shown in Fig. 2, TRAAK mRNA expression in the spinal cord was significantly higher in the DO rats than in the sham-operated rats (1.886 ± 0.710 versus 0.790 ± 0.679, *P* < 0.05). Nevertheless, no significant differences in the bladder were observed between the two groups (0.031 ± 0.017 versus 0.027 ± 0.019, *P* > 0.05).

### TRAAK protein expression

**Fig. 3:**
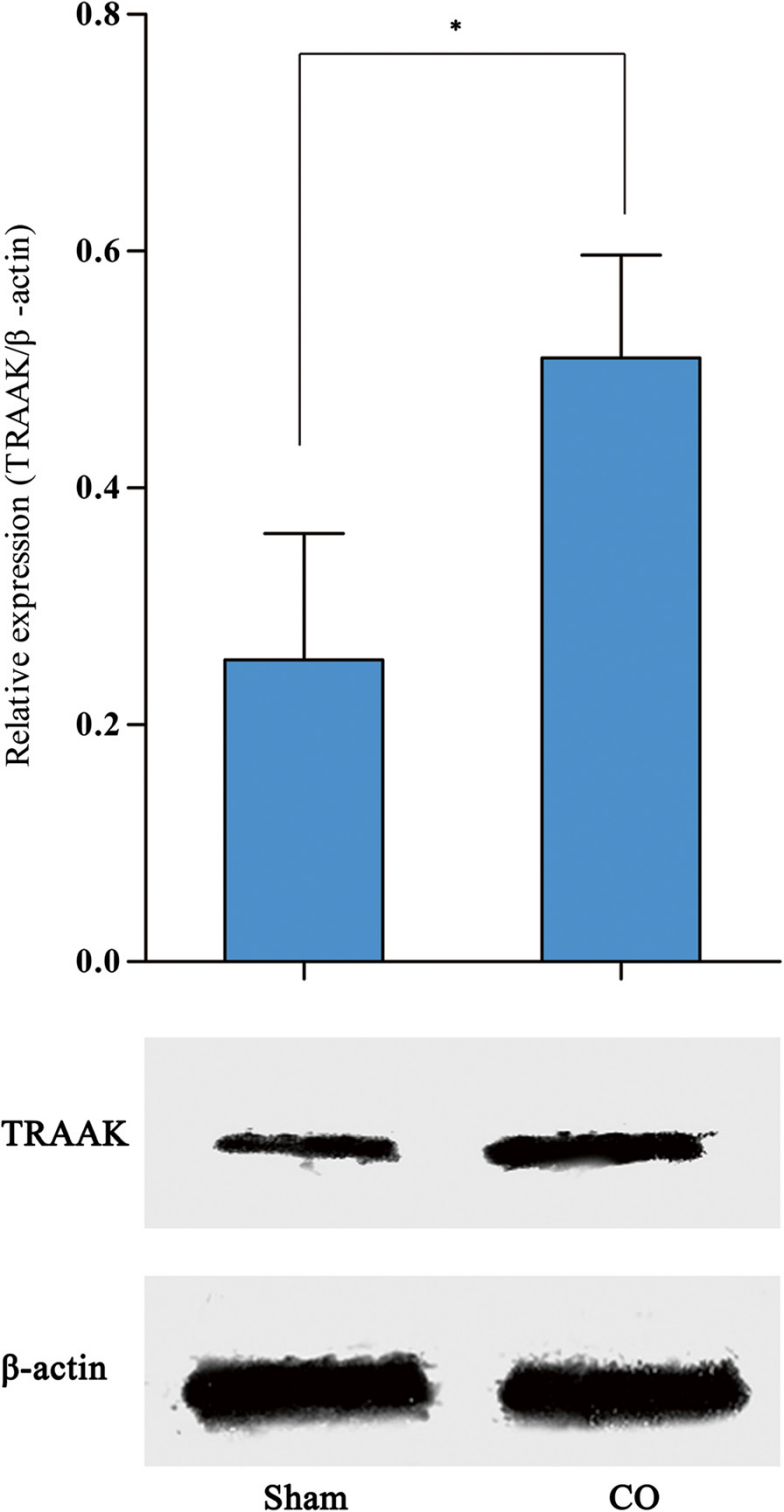
TRAAK protein expression in the spinal cord of DO and sham-operated groups. Data are presented as means (SD) of TRAAK/β-action. DO rats *n* = 4; Sham-operated rats *n* = 4. *P < 0.05

**Fig. 4:**
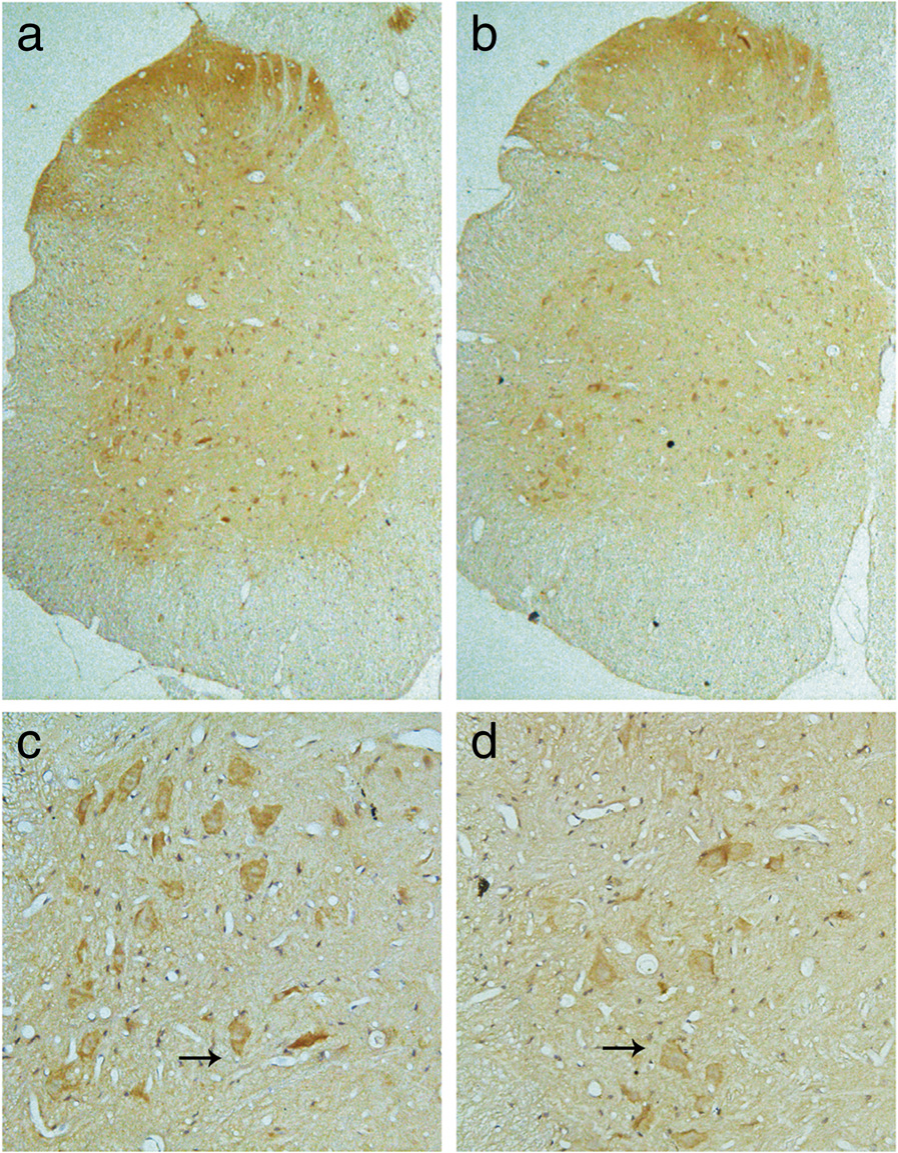
TRAAK protein detected by immunohistochemistry. **a**, **c** Section from DO rats (magnification: [a] × 50; [c] × 200); **b**, **d** Section from sham-operated rats (magnification: [b] × 50; [d] × 200). Black arrows indicate immunopositive neurons

Western blotting showed that TRAAK protein expression in the L6-S1 spinal cord of DO rats was significantly higher than that in sham-operated rats (0.510 ± 0.087 versus 0.255 ± 0.107, *P* < 0.05), which corresponded to the RT-PCR results (Fig. 3). Immunohistochemical staining showed that the increased TRAAK protein expression was mainly located in the gray matter of the spinal cord, including the dorsal horn and ventral horn. The immunoreactive signals of TRAAK in both regions were higher in the DO rats than in the sham-operated rats (Fig. 4).

## Discussion

DO is a urodynamic observation characterized by involuntary detrusor contractions during the filling phase [13]. It is associated with age, PBOO or benign prostatic enlargement [2]. However, the pathogenesis of PBOO-related DO has not been fully elucidated [14, 15]. Ion channels are associated with resting membrane potential and cellular excitability [5]. Numerous studies have shown alteration of ion channel expression in detrusor myocytes of rats with DO, which is thought to be a causative agent of bladder dysfunction [3, 4]. However, most of those studies focused on the bladder. Bladder function is partly controlled by the peripheral and central nervous systems; therefore, investigating the physical status of neurons will greatly help to elucidate the mechanism of DO.

PBOO rats are the most classical animal models for exploring the mechanism of DO [16, 17]. In the present study, a PBOO rat model was successfully established by tying a ligature around the bladder neck with a small plastic tube (O.D. 1.0 mm) placed as a catheter via the urethral orifice. Six weeks after the establishment of PBOO, NVCs of the detrusor muscle were detected by cystometry. According to the definition of DO proposed by the International Continence Society, there is no lower limit for the amplitude of an involuntary detrusor contraction for DO [13]. Therefore, we did not set a limit for the amplitude of NVCs in this study when classifying the DO group. The incidence rate of NVCs in the PBOO rats was approximately 62.5 %, which was similar to rates reported in previous studies [4]. Rats displaying NVCs were classified as DO group for further study. In the present study, the bladder capacity was significantly higher in the DO rats than in the sham-operated rats, which was consistent with the finding of previous papers with similar animal models [8, 16]. As we know, in clinic, DO is often observed in patients with bladder outlet obstruction. Some of these patients may show increased residual urine and higher capacity in urodynamic studies. The higher bladder capacity may be caused by chronic overdistension of the bladder subsequent to obstruction of bladder outlet.

TRAAK, TREK-1 and TREK-2 are protein members of the TREK subfamily of the 2P-domain K^+^ channel and mechano-gated K^+^ channel family. This subfamily of K^+^ channels is commonly believed to include inward-rectifying potassium channels that are associated with resting membrane potential and cellular excitability [9]. The pathophysiologic mechanisms of DO are associated with various kinds of ion channels, such as T-type calcium, calcium-activated K^+^ and Cl^−^ channels [3, 4]. Salah A. Baker firstly reported the downregulation of the TREK-1 channel in detrusor myocytes of DO rats with PBOO, and this downregulation was thought to be associated with over-excitability of the detrusor smooth muscle [11]. In the present study, both the mRNA and protein levels of the TRAAK channel were found to be up-regulated in the L6-S1 spinal cord of DO rats. Immunohistochemical staining showed increased expression of the TRAAK channel in the dorsal horn and ventral horn of the spinal cord, which represent the location of sensory neurons and motor neurons, respectively. Upregulation of the TRAAK channel in the neurons in the L6-S1 spinal cord decreased their excitability. This upregulation may not only decrease the excitability of sensory neuron, but may also suppress neural motor output, which wound subsequently suppress NVCs of the detrusor muscle in the bladder. According to the results of this study, we hypothesize that upregulation of the TRAAK channel in the spinal cord after chronic PBOO exerts a protective effect against DO of the bladder. Upregulation of the TRAAK channel in the spinal cord may be the result of bladder inflammation caused by chronic PBOO, as there is evidence that bladder inflammation affects rat spinal neurons [18]. Upregulation of the TRAAK channel in the spinal cord may also be associated with negative feedback in response to the long-lasting stimulus of the overdistended bladder.

TRAAK mRNA expression in the bladder was not significantly different between the DO and control groups, which was consistent with a previous study that found no alteration of TRAAK expression in bladder of rats with CBOO [10]. As the TRAAK channel was mainly found in the central nervous system [19, 20], the TRAAK channel may not be involved in the myogenic mechanism of DO after PBOO.

There are some limitations of this study. First, cystometry was performed in anesthetized rats. Anesthetization may, to some extent, preclude information on active mictrurition compared to the normal physiological state. Second, the present study did not include a TRAAK channel blocker group due to lack of TRAAK channel-specific blockers that can be used in vivo.

## Conclusion

In conclusion, the TRAAK channel was up-regulated in the spinal cord of rats with DO induced by chronic PBOO, which is thought to decrease the excitability of the neurons in the central nervous system and may exert a protective effect against bladder dysfunction in DO rats.

## Abbreviations

CBOO: Complete bladder outlet obstruction
DO: Detrusor overactivity
NVCs: Nonvoiding contractions
PBOO: Partial bladder outlet obstruction
RT-PCR: Real-time polymerase chain reaction
TREK: TWIK-related K^+^ channel
TRAAK: TWIK-related arachidonic acid-activated K^+^ channel
TWIK: The weak inward rectifying K^+^ channel
